# Wer fühlt sich exkludiert? Zur zeitdiagnostischen Verwendung des Konzepts der sozialen Exklusion

**DOI:** 10.1007/s11577-021-00802-7

**Published:** 2021-10-12

**Authors:** Audrey Djouadi, Jörg Rössel, Alexander Seifert

**Affiliations:** 1 Soziologisches Institut, Universität Zürich, Andreasstrasse 15, 8050 Zürich, Schweiz; 2 Hochschule für Soziale Arbeit FHNW, Riggenbachstrasse 16, 4600 Olten, Schweiz

**Keywords:** Zeitdiagnose, Bildung, Staatsbürgerschaft, Altersgruppen, Exklusionsempfinden, Analysis of current societal trends, Education, Citizenship, Age groups, Sense of exclusion

## Abstract

Der Begriff der sozialen Exklusion hat in den Sozialwissenschaften eine erstaunliche Karriere erfahren. Im Mittelpunkt des Beitrags steht die empirische Untersuchung der zeitdiagnostischen Verwendung des Konzepts. Aus dieser leiten wir vier Thesen ab, die in diesem Beitrag mit dem Fokus auf das Exklusionsempfinden empirisch geprüft werden: Erstens, dass aufgrund der Prozesse des ökonomischen Strukturwandels größere Bevölkerungsgruppen von sozialer Exklusion in mehreren Dimensionen (Arbeitslosigkeit, Armut, soziale Isolation) betroffen sind, die bei diesen in einem subjektiven Exklusionsempfinden kulminieren. Damit wird unterstellt, dass soziale Exklusion zur Hauptspannungslinie der gegenwärtigen Gesellschaft geworden ist. Zweitens wird angenommen, dass soziale Exklusion nicht eindeutig in klassischen sozialstrukturellen Kategorien zu verorten ist, sondern in breite Teile der Gesellschaft diffundiert ist. Drittens wird sozioökonomischer Prekarisierung und sozialer Isolation eine zentrale Rolle für die Entstehung eines subjektiven Exklusionsempfindens zugesprochen. Hier wird allerdings, viertens, vermutet, dass dieses vermittelt über die subjektive Wahrnehmung der objektiven Lage auf das Exklusionsempfinden wirkt. Wir prüfen diese Thesen des Konzepts auf der Basis von Umfragedaten, wobei wir das Exklusionsempfinden als abhängige Variable verwenden. Dabei wird deutlich, dass erstens soziale Exklusion nicht in weite Teile der Gesellschaft diffundiert ist und damit keineswegs als Hauptspannungslinie der Gesellschaft betrachtet werden kann, zweitens sich ein erhöhtes Exklusionsempfinden in unterschiedlichen, aber klar benennbaren sozialen Gruppen feststellen lässt. Darüber hinaus zeigen unsere Analysen, dass das subjektive Exklusionsempfinden sowohl in sozialer Isolation als auch in sozioökonomischer Prekarisierung begründet ist, allerdings deutlich vermittelt über deren subjektive Wahrnehmung.

## Abstract (en)

The concept of social exclusion has had an astonishing career in the social sciences. The focus of this paper is an empirical investigation of the use of this concept to analyze current societal trends. From this theoretical perspective we derive four theses, which are empirically tested in this paper with a focus on the perception of exclusion: First, that because of the processes of economic structural change, larger population groups are affected by social exclusion in several dimensions (unemployment, poverty, social isolation), culminating in a subjective sense of exclusion among them. Thus, it is assumed that social exclusion has become the main social cleavage in contemporary society. Second, it is assumed that social exclusion cannot be clearly located in classical sociostructural categories but has diffused into broad segments of society. Third, socioeconomic precariousness and social isolation are thought to play a central role in the emergence of a subjective sense of exclusion. Here, and fourth, it is assumed, however, that this impacts on the sense of exclusion via the subjective perception of the objective life conditions. We test these theses derived from this theoretical perspective on the basis of survey data, using the sense of exclusion as a dependent variable. It becomes clear that, first, social exclusion has not diffused into large parts of society and thus can by no means be regarded as the main social cleavage in society; and second, an increased sense of exclusion can be found in different but clearly identifiable social groups. Moreover, our analyses show that the subjective sense of exclusion is rooted in both social isolation and socioeconomic precariousness, albeit clearly mediated by their subjective perception.

## Einleitung

Der Begriff der sozialen Exklusion hat in den Sozialwissenschaften eine erstaunliche Karriere erfahren. Finden sich in den 1980er-Jahren erst wenige Veröffentlichungen, in denen das Konzept überhaupt auftaucht, so geht die Anzahl der Publikationen gegenwärtig in die Hunderte.^1^ Allerdings verbergen sich hinter dem Wort ausgesprochen unterschiedliche theoretische Konzeptualisierungen, die sowohl umfassende gesellschaftsheoretische Perspektiven als auch spezifische empirische Forschungen über die Exklusion bestimmter sozialer Gruppen einschließen.^2^ Bei den gesellschaftstheoretischen Ansätzen sind einerseits die Systemtheorie und andererseits die zeitdiagnostische Verwendung des Konzepts der Exklusion hervorzuheben. In der systemtheoretischen Verwendung des Begriffs geht es vor allem um die Frage, wie Personen innerhalb der Teilsysteme der funktional differenzierten modernen Gesellschaft adressiert werden. Die zeitdiagnostische Verwendung des Begriffs, die auch in der öffentlichen Diskussion auf große Resonanz gestoßen ist, sieht soziale Exklusion als ein Konzept, das zentrale Merkmale von Gegenwartsgesellschaften erfasst. Im Mittelpunkt unseres Beitrags soll insbesondere die zeitdiagnostische Verwendung des Konzepts stehen, deren zentrale Thesen empirisch geprüft werden.

Im Kern behaupten die zeitdiagnostischen Verwendungsweisen des Begriffs als zentrale Thesen, dass erstens soziale Exklusion die Hauptkonfliktlinie von gegenwärtigen Gesellschaften darstellt (Bude 1998, 2004; Castel 2000; Kronauer 2010; Kronauer und Häussermann 2016). Damit träte sie an die Stelle des in den kapitalistischen Industriegesellschaften des 19. und 20. Jahrhunderts bis in die 1970er-Jahre dominanten Klassenkonflikts zwischen Arbeit und Kapital. Insbesondere Castel (2000) und Kronauer (2010) sprechen statt von einer Konfliktlinie von „der“ sozialen Frage einer Gesellschaft, die den Kristallisationspunkt von Auseinandersetzungen über die gesellschaftliche Zukunft darstellt. War dies im 20. Jahrhundert die Arbeiterfrage, so ist dies im beginnenden 21. Jahrhundert die Frage der sozialen Exklusion. Soziale Exklusion wird hier nicht als bloße sozioökonomische Marginalisierung definiert, sondern als eine Verkettung von Exklusionsprozessen in verschiedenen gesellschaftlichen Dimensionen: Neben den Ausschluss aus dem Erwerbsleben und die damit verbundene Lebenslage der ökonomischen Armut tritt die zunehmende soziale Isolation, die sich auch in Form der räumlichen Segregation von exkludierten Gruppen materialisiert. Soziale Exklusion wird also als ein mehrdimensionaler Prozess des sozialen Ausschlusses definiert. Diese Kette wird von zahlreichen Autoren (Kronauer 2010; Gallie et al. 2003; Castel 2000) in Form eines selbstverstärkenden Zirkels konzeptualisiert. In der Zeitdiagnose der sozialen Exklusion wird darüber hinaus als zweite zentrale These – vergleichbar der Individualisierungstheorie – unterstellt, dass Prozesse der sozialen Exklusion sozialstrukturell, insbesondere in der Klassenhierarche, nicht klar zu verorten sind. Bude zieht hier Begriffe wie Querkategorie und transversale Kategorie heran (Bude 1998, 2004). Wie Bude und Lantermann (2006) zusammenfassen, kann soziale Exklusion allerdings nicht als rein objektivistisches Konzept verstanden werden. Die miteinander verbundenen objektiven Dimensionen der Ausgrenzung, also sozioökonomische Marginalisierung und soziale Isolation, sind die Grundlage für daraus resultierende Gefühle des Nichtdazugehörens, des nicht in die Gesellschaft Eingebundenseins. So wie sich die Klassenspaltung der kapitalistischen Industriegesellschaft in einem Klassenbewusstsein geäußert hat, so resultieren die mehrdimensionalen Prozesse der sozialen Exklusion in einem subjektiven Gefühl des Ausgeschlossenseins – dem Exklusionsempfinden. Diesen Zusammenhang zwischen objektiver Lage einerseits und subjektivem Exklusionsempfinden andererseits werden wir in unserem Beitrag als dritte These des zeitdiagnostischen Konzepts untersuchen, weshalb wir das Exklusionsempfinden als abhängige Variable betrachten müssen. Bude und Lantermann (2006) gehen in ihrem Modell noch einen Schritt weiter und behaupten als vierte These, dass die objektive Lage erst über ihre subjektive Wahrnehmung und psychologische Verarbeitung das Exklusionsempfinden affiziert. Die hier skizzierten vier Thesen der zeitdiagnostischen Verwendung des Exklusionskonzepts prüfen wir in unserem Beitrag empirisch anhand der Verbreitung des Exklusionsempfindens in der Bevölkerung der Schweiz.

Dieser zeitdiagnostischen Vorstellung von sozioökonomisch bedingter Exklusion als zentraler Spannungslinie gegenwärtiger Gesellschaften, die sozialstrukturell unspezifisch ist, steht eine Vielzahl von Studien gegenüber, die soziale Exklusionsprozesse für spezifische Bevölkerungsgruppen rekonstruiert und nachweist. So zeigt Groh-Samberg (2014) beispielsweise, dass das Risiko der Verfestigung von Armut nachhaltig klassenspezifisch geprägt ist, also ganz bestimmte Bevölkerungsgruppen betrifft. Betrachtet man dagegen die Diskussion über die Diskriminierungswahrnehmungen von ausländischen Personen und Personen mit Migrationshintergrund (Diehl et al. 2021), so rücken hier ganz andere Mechanismen der sozialen Exklusion in den Vordergrund. Untersuchungen über ältere Menschen wiederum zeigen, dass diese sich aufgrund ihres Gesundheitszustands, aber zum Teil auch aufgrund der aktuellen technologischen Entwicklungen im Bereich der Digitalisierung ausgeschlossen fühlen (Kristensen et al. 2019; Seifert et al. 2018). Schließlich kann für den von uns untersuchten schweizerischen Fall auch festgehalten werden, dass die kleineren Sprachgruppen (Französisch, Italienisch) in diesem mehrheitlich deutschsprachigen Land sich durch die Deutschschweizer majorisiert fühlen (Kriesi et al. 1996), hier der Situation der Ostdeutschen im vereinigten Deutschland nicht unähnlich (Schneickert et al. 2019). Die hier angeführten Beispiele zeigen, dass Exklusionsprozesse im Gegensatz zur zweiten These des zeitdiagnostischen Konzepts in bestimmten, sozialstrukturell definierten Gruppen verstärkt auftreten können. Im Anschluss an diese Beispiele werden wir im Beitrag den empirischen Fokus auf Bildungsgruppen als klassische, vertikale Dimension der Sozialstruktur, Altersgruppen, Ausländer und Inländer sowie die 3 Sprachgruppen in der Schweiz richten und prüfen, ob das Exklusionsempfinden entlang dieser Variablen sozial strukturiert ist (vgl. Schneickert et al. 2019).^3^

In unserem Beitrag wollen wir also die vier skizzierten Thesen des zeitdiagnostischen Exklusionskonzepts mit einem Fokus auf die wahrgenommene soziale Exklusion empirisch untersuchen. Unter Verwendung der von Bude und Lantermann (2006) entwickelten Skala des Exklusionsempfindens soll erstens geprüft werden, ob die erste zentrale These von der sozialen Exklusion als Hauptkonfliktlinie der Gesellschaft empirische Plausibilität aufweist. Zweitens soll untersucht werden, ob die wahrgenommene soziale Exklusion entsprechend der zweiten zentralen These des zeitdiagnostischen Konzepts ein entgrenztes Phänomen ist, das sich sozialstrukturell kaum eingrenzen lässt oder ob es sich um klar abgrenzbare soziale Gruppen handelt, die sich überdurchschnittlich häufig exkludiert fühlen. Zudem soll auf dieser Grundlage der dritten These folgend auch betrachtet werden, ob Prozesse der sozioökonomischen Marginalisierung und der sozialen Isolation mit dem Exklusionsempfinden kovariieren, wobei wir entsprechend der vierten These auch die subjektive Wahrnehmung der vorhandenen Ressourcen und Problemlagen als vermittelnde Konzepte empirisch berücksichtigen können. Für diese empirische Untersuchung greifen wir auf eine relativ aktuelle bevölkerungsrepräsentative Umfrage aus der Schweiz zurück, die zur Jahreswende 2019/20 durchgeführt wurde. Im folgenden Abschn. 2 werden wir die im Mittelpunkt unseres Artikels stehende zeitdiagnostische Lesart des Begriffs der sozialen Exklusion und den Forschungsstand zur Exklusion entlang von Bildung, Alter, Staatsbürgerschaft und Sprachgruppe vorstellen, um auf dieser Grundlage empirisch prüfbare Fragestellungen zu begründen. Die verwendeten Daten und empirischen Methoden werden in Abschn. 3 erläutert, um dann in Abschn. 4 die empirischen Ergebnisse darzulegen. Unsere Daten zeigen, dass das zeitdiagnostische Konzept der sozialen Exklusion mit seinen zentralen Thesen nur begrenzt geeignet ist, um die zentralen Spannungslinien von Gegenwartsgesellschaften zu erfassen. Eine Wahrnehmung von sozialer Exklusion ist erstens nur in kleinen Teilen der Bevölkerung verbreitet, die zweitens klar sozialstrukturell zu verorten sind. Die dritte und vierte These erweisen sich demgegenüber als empirisch haltbare Ansätze für die Analyse der Verursachungszusammenhänge des Exklusionsempfindens.

## Theoretischer Rahmen und Forschungsstand

### Soziale Exklusion als Zeitdiagnose

Sich zu Gruppen und zur Gesellschaft zugehörig zu fühlen, ist ein menschliches Grundbedürfnis (Baumeister und Leary 1995). Wenn dieses nicht erfüllt wird, führt dies laut psychologischen Forschungen zu kognitiven Einschränkungen, selbstschädigendem Verhalten und zu emotionalem Stress (Twenge et al. 2002). Daher betonen Bude und Lantermann (2006, S. 234) die zentrale Bedeutung der subjektiven Wahrnehmung für das Exklusionskonzept. Wenn lediglich eine objektive Benachteiligung vorliegt, sprechen die beiden Autoren von Marginalisierung, aber nicht von Exklusion. Für die Analyse von Exklusionsprozessen reicht damit der Fokus auf die objektive Lage nicht aus, sondern als abhängige Variable muss betrachtet werden, ob Menschen sich tatsächlich als exkludiert wahrnehmen. Auch wenn die subjektive Wahrnehmung von sozialer Exklusion von entscheidender Bedeutung ist, beruht diese auf einem komplexen, mehrdimensionalen Prozess, der im Folgenden erläutert werden soll.

Übereinstimmend sehen zahlreiche Vertreter eines zeitdiagnostischen Exklusionskonzepts dieses als mehrdimensional an. Soziale Exklusion in einem umfassenden Verständnis ist danach gekennzeichnet durch den Ausschluss aus dem Erwerbssystem, ökonomische Prekarität und Armut, soziale Isolation und räumliche Segregation und die subjektive Wahrnehmung von Exklusion sowie den darauf basierenden Rückzug aus der Gesellschaft (Gallie et al. 2003; Kronauer und Häussermann 2016; Kronauer 2010; Castel 2000; Burchardt et al. 2002; Bude 1998; Ludwig-Mayerhofer 2009). Den makrostrukturellen Hintergrund für die Verbreitung dieser Prozesse in der Gesellschaft bilde die Transformation der kapitalistischen Wirtschaftsform seit den 1970er-Jahren, die durch eine Flexibilisierung und Prekarisierung von Arbeitsverhältnissen, zunehmende Arbeitslosigkeit, Armut und einen liberalen Umbau des Wohlfahrtsstaats gekennzeichnet sei (Kronauer und Häussermann 2016; Kronauer 2010; Ludwig-Mayerhofer 2009). Damit sei an die Stelle der bisherigen Klassenspaltung der Gesellschaft als zentraler sozialer Frage die Unterscheidung zwischen den dazu Gehörenden und den sozial Exkludierten als hauptsächlicher Spannungslinie der Gesellschaft getreten (Bude 1998; Castel 2000; Kronauer 2010; Ludwig-Mayerhofer 2009).^4^ Dies stellt aus unserer Sicht die erste zentrale These des zeitdiagnostischen Konzepts dar. Diese neue Bruchlinie sei nicht mehr an traditionelle Sozialstrukturen, insbesondere Klassenstrukturen gekoppelt (Bude 1998, S. 373, 2004; Kronauer 2010). Insbesondere Bude wirft der traditionellen Sozialstrukturanalyse vor, dass ihr Festhalten an klassischen sozialstrukturellen Kategorien weitverbreitete und öffentlich diskutierte Empfindungen der Exklusion oder der Exklusionsgefährdung nicht erfassen könne (Bude 2004, S. 3). Er spricht von einer Querkategorie oder einer transversalen Kategorie, die sich durch klassische Konzepte der Sozialstrukturanalyse nicht mehr fassen ließe (Bude 1998, 2004), wobei das Exklusionsempfinden immer größere Teile der Bevölkerung erfasse und damit auch als eine Art „spill over effect“ (Lengfeld und Hirschle 2009) bis in die Mittelschichten hineinwirke. Diese sozialstrukturelle Unspezifizität der sozialen Exklusion ist in unserer Lesart die zweite These des zeitdiagnostischen Konzepts der sozialen Exklusion. Die behaupteten Zusammenhänge zwischen objektiven Prozessen der Marginalisierung und der sozialen Isolation einerseits und dem subjektiven Exklusionsempfinden andererseits stellen eine dritte These des zeitdiagnostischen Konzepts dar. In der Untersuchung von Bude und Lantermann (2006) wird als vierte hier betrachtete These, deutlich, dass die faktische soziale Lage von Personen ihr Exklusionsempfinden beeinflusst, dies aber vermittelt über komplexe psychologische Prozesse der Verarbeitung von objektiver Marginalisierung. Diese schließen neben den internen, psychologischen Ressourcen einer Person (Kohärenzsinn, Unbestimmtheitsorientierung) die Bewertung der gegenwärtigen und zukünftigen Ressourcenlage ein.

Damit stellt sich das zeitdiagnostische Konzept der Exklusion als komplexe These über den gesellschaftlichen Wandel und die strukturelle Situation von benachteiligten Personen in westlichen Gesellschaften dar, die wir im Folgenden empirisch einordnen wollen, wobei wir in unserer Darstellung vor allem die Literatur zu Deutschland und zur Schweiz betrachten werden, da die hier diskutierte Variante des zeitdiagnostischen Konzepts der sozialen Exklusion vor allem auf den deutschsprachigen Kontext fokussiert ist.

Betrachtet man die in der Exklusionsthese behaupteten strukturellen Entwicklungen einerseits, die Verknüpfungen zwischen diesen Prozessen andererseits, so ergibt sich ein gemischtes Bild. Seit den 1970er-Jahren hat die Arbeitslosigkeit in den EU-Ländern im Mittel deutlich zugenommen, wobei allerdings in Deutschland die Arbeitslosenquote seit den frühen Jahren des 21. Jahrhunderts wieder auf ein Niveau von 4 % abgesunken ist, und auch in der Schweiz fluktuiert die Arbeitslosigkeit seit den 1990er-Jahren um ca. 3 % (Sheldon 2013). Allerdings hat seit den 1990er-Jahren in Deutschland der Anteil von atypischen Arbeitsverhältnissen zugenommen, seien dies befristete Beschäftigungen, Zeit- und Leiharbeit, geringfügig Beschäftigte und Soloselbständige, die häufig nur geringe Einkommen erzielen (Crössmann und Günther 2018). Damit verbunden ist seit den 1970er-Jahren eine Zunahme von Einkommensungleichheit (Gini-Koeffizient) und Armut (Medianeinkommen), die allerdings nicht stetig verlaufen sind (Rössel 2009; Kott 2018; Goebel und Krause 2018). Die Armutsgefährdungsquote in Deutschland liegt in den vergangenen Jahren um 16 %, in der Schweiz unwesentlich tiefer um 15 % (Kott 2018; Bundesamt für Statistik 2020). Im internationalen Vergleich zeigen sich sowohl für die Herausbildung prekärer Arbeitsverhältnisse als auch für die Entwicklung der Armutsquote je nach geographischer Region deutlich unterschiedliche Entwicklungen (Fritsch und Verwiebe 2018; Kalleberg und Vallas 2018). Entsprechend der Exklusionsthese kann also seit den 1970er-Jahren die Herausbildung eines Bevölkerungssegments festgestellt werden, das unter ökonomisch prekären Bedingungen lebt, seien dies Erwerbslosigkeit, atypische Beschäftigungen oder Armutsgefährdung, auch wenn diese nicht den Umfang erreicht, der in den zitierten Zeitdiagnosen insinuiert wird. Allerdings kann auf der Basis dieser Angaben nicht geschlussfolgert werden, dass es sich hier um eine über die Zeit stabile Bevölkerungsgruppe handelt. Die dynamische Armutsforschung hat den Fokus auf die Tatsache gerichtet, dass Menschen den Armutsstatus nicht zwingend dauerhaft aufweisen, sondern zwischen Phasen der Armut und der Nichtarmut im Lebensverlauf wechseln können (Giesselmann und Vandecasteele 2018). Diese Forschung zeigt nun in der Tat für Deutschland eine zunehmende zeitliche Verfestigung von Armut (Giesselmann und Vandecasteele 2018; Groh-Samberg 2014). Diese verfestigte, von Armut betroffene Gruppe macht nach Groh-Samberg (2014) zwischen 10 (alte Bundesländer) und 12 % (neue Bundesländer) der Bevölkerung in Deutschland aus. Dies kann für die Schweiz nicht in gleichem Maße festgestellt werden. Der Anteil von Personen, die vier aufeinanderfolgende Jahre armutsgefährdet waren, lag dort immer unter 5 % (Bundesamt für Statistik 2018). Auch für die von prekären Beschäftigungsverhältnissen betroffene Bevölkerung konstatieren Promberger et al. (2018), dass rund ein Achtel der deutschen Bevölkerung dauerhaft in prekären Arbeitsverhältnissen beschäftigt ist. Böhnke et al. (2016) stellen allerdings fest, dass mit dieser prekären Erwerbslage, die insbesondere Frauen in Familienhaushalten betrifft, keine überdurchschnittlichen Armutsrisiken verbunden sind. In der Schweiz liegt dagegen der Anteil von Personen in atypisch-prekären Beschäftigungsverhältnissen auf einem deutlich niedrigeren Niveau um 2,5 %, zudem handelt es sich überwiegend nicht um ein dauerhaftes Verbleiben in solchen Beschäftigungsverhältnissen (Ecoplan 2017). Während also der Umfang der armutsgefährdeten Bevölkerung in der Schweiz und in Deutschland übereinstimmend bei ca. 15 % liegt, ist der Anteil von Personen mit prekärer Beschäftigung in Deutschland deutlich höher als in der Schweiz. Zudem handelt es sich sowohl bei den armutsgefährdeten als auch bei den prekär Beschäftigten in Deutschland sehr viel stärker um eine über die Zeit hinweg verfestigte Gruppe. Schließlich kann aber für diese Gruppen festgehalten werden, dass sie im Gegensatz zur zeitdiagnostischen Exklusionsthese immer stärker durch klassische sozialstrukturelle Merkmale beschrieben werden können (Giesselmann und Vandecasteele 2018; Groh-Samberg 2014; Böhnke et al. 2016; Hümberlin und Fritschi 2016; Vandecasteele 2011), insbesondere Bildung als Klassenmerkmal und Geschlecht strukturieren Armut und prekäre Beschäftigung in hohem Maße. Es kann also im Gegensatz zur zeitdiagnostischen Verwendung des Exklusionskonzepts nicht von entgrenzten Risiken der Marginalisierung gesprochen werden.

Das Konzept der sozialen Exklusion ist mehrdimensional angelegt, es wird also nicht nur die Herausbildung einer in sozioökonomischer Hinsicht prekären oder marginalen Bevölkerungsgruppe diagnostiziert, sondern darüber hinaus werden eine zunehmende soziale Isolation dieser Menschen und ihre zunehmende räumliche Konzentration vermutet. Dies führe zu einer sozialen Exklusion in mehreren Dimensionen, die sich dann wiederum in einer Art Teufelskreis gegenseitig verstärken. Wir betrachten zunächst soziale Isolation und Einsamkeit. Eckhard (2018) kann für Deutschland mit den Daten des Sozio-oekonomischen Panels einen Bevölkerungsanteil zwischen 3 und 10 % feststellen, der sozial isoliert ist, wobei im Zeitverlauf keine klare Zunahme zu verzeichnen ist. Im Gegensatz dazu betrachten Böger et al. (2017) den Anteil von Personen, die sich einsam fühlen. Sie berichten auf der Basis des Deutschen Alterssurveys für Personen über 40 Jahre zwischen 1996 und 2014 eine leichte Abnahme des Anteils von solchen Personen (von 10,7 auf 8,9 %). Für die Schweiz liegen lediglich Informationen über das Einsamkeitsgefühl und nicht über faktische soziale Isolation vor. Diese zeigen für den Zeitraum von 2002–2017 relativ konstant einen Wert von 8 % der Bevölkerung, die sich einsam fühlen.^5^ Im europäischen Vergleich liegen die für Deutschland und die Schweiz genannten Werte auf einem vergleichsweise niedrigen Niveau (D’Hombres et al. 2021). Insgesamt wird deutlich, dass der Anteil der sozial isolierten und einsamen Personen an der Bevölkerung kleiner ist als der Anteil der armutsgefährdeten Menschen und keine zunehmende Tendenz aufweist.

Abschließend soll noch die Entwicklung der sozialen Segregation betrachtet werden. Im Kontext der zeitdiagnostischen Verwendung des Exklusionsbegriffs wird eine steigende soziale Segregation angenommen, die zu einer Verstärkung der Ausgrenzung von exkludierten Gruppen führe (Kronauer und Häussermann 2016). Helbig und Jähnen (2018) können aufzeigen, dass es seit den frühen 1980er-Jahren in Deutschland in der Mehrzahl der Großstädte tatsächlich zu einer zunehmenden Segregation von armen Personen gekommen ist. Dagegen hat die ethnische Segregation tendenziell eher abgenommen. Für die Schweiz liegen nur vereinzelte Studien zur sozialen und ethnischen Segregation vor (Heye und Leuthold 2006; Schaerer und Baranzini 2009). Diese deuten weder auf eine Zunahme der ethnischen noch der sozialen Segregation hin. Die in den vorliegenden deutschen und schweizerischen Studien berichteten Segregationsindizes um 0,2–0,25 liegen im internationalen Vergleich auf einem niedrigen Niveau (Musterd 2005; Musterd et al. 2017).

Die zeitdiagnostische Version der Exklusionsthese unterstellt weiterhin eine Verknüpfung zwischen den verschiedenen Dimensionen der sozialen Exklusion. Es ist allerdings offensichtlich, dass bei einer gleichzeitigen Betrachtung von Erwerbslosigkeit, Armutsgefährdung, sozialer Isolation und Segregation das Muster der objektiven sozialen Exklusion auf immer kleinere Gruppen zutreffen muss, da nur ein Teil der armutsgefährdeten Personen auch sozial oder räumlich isoliert sind. Dies wird auch durch Studien bestätigt, die das gleichzeitige Auftreten mehrerer Exklusionsdimensionen oder deren Zusammenhang untersucht haben (Burchardt et al. 2002; Gallie et al. 2003; Bundesamt für Statistik 2019). Für das Jahr 2016 dokumentiert das Schweizer Bundesamt für Statistik für 3,6 % der Gesamtbevölkerung in der Schweiz objektive und subjektive Mehrfachbenachteiligungen, wobei die Anteile unter Erwerbslosen, Einkommensschwachen und Ausländern besonders hoch waren (Bundesamt für Statistik 2019). Es handelt sich also bei den mehrfach exkludierten sozialen Personen, die dem zeitdiagnostischen Modell der Exklusion entsprechen, um ein sehr kleines Bevölkerungssegment.

Allerdings wird in der zeitdiagnostischen Verwendung des Exklusionsbegriffs nicht allein die objektive Seite der Exklusion betrachtet, sondern auch ihre subjektive Wahrnehmung, die hier im empirischen Fokus des Beitrags steht. Bude und Lantermann (2006) machen deutlich, dass auch mehrfach benachteiligte Personen das Gefühl haben können, ihr Leben zu meistern und sich nicht ausgeschlossen zu fühlen. Exklusion ist aus ihrer Sicht letztlich daran geknüpft, dass Menschen sich tatsächlich als nicht dazugehörig empfinden und sich nicht mehr im „Drinnen“ der Gesellschaft sehen, sondern im „Draußen“. Damit wird die subjektive Wahrnehmung von Ausgeschlossensein zum entscheidenden Kriterium für die Anwendung des Exklusionsbegriffs, weshalb unser Beitrag auf das Exklusionsempfinden fokussiert. Die beiden Autoren haben daher ein empirisches Instrumentarium für dessen Messung entwickelt. Auf dieses greifen wir auch in unserer Studie zurück. Leider berichten Bude und Lantermann (2006) in ihrer Studie nicht den Anteil von Personen, die sich subjektiv als exkludiert wahrnehmen. Daher kann auf dieser Basis nicht eingeschätzt werden, ob und in welchem Umfang die zeitdiagnostische Exklusionsthese für Deutschland zutrifft. Betrachtet man als Proxy die Sorge vor dem Arbeitsplatzverlust, so berichten Lengfeld und Hirschle (2009) für Westdeutschland zwischen 1984 und 2007 tatsächlich eine deutliche Zunahme dieser Sorge, die allerdings stark nach sozialen Klassen gegliedert ist. Seit ungefähr 2005 nimmt die Sorge um den Arbeitsplatz wiederum massiv ab (Lengfeld 2019). Böger et al. (2017) berichten auf der Grundlage des Deutschen Alterssurveys für das Jahr 2014 einen Anteil von 6,4 % der über 40-Jährigen, die sich als exkludiert wahrnehmen, wobei dieser Anteil stark mit dem sozioökonomischen Status variiert. In dieser Studie wurde allerdings nicht das von Bude und Lantermann (2006) entwickelte Konzept verwendet. Schneickert et al. (2019) zeigen, dass in Deutschland Menschen insgesamt viel Wertschätzung und wenig Geringschätzung erfahren. Diese Erfahrungen sind aber deutlich sozial strukturiert. Damit liegen also Hinweise zu verwandten Konzepten vor, aber belastbare Angaben über die Verbreitung des Exklusionsempfindens in der Gesamtbevölkerung gibt es bisher nicht. Diese Lücke soll die vorliegende Studie schließen.

### Soziale Exklusion in sozialstrukturell definierten Gruppen

In der zeitdiagnostischen Variante der Exklusionsthese wurde vor allem ein kausaler Mechanismus für die Entstehung von Exklusionsempfinden in den Vordergrund gestellt, den wir hier als dritte These untersuchen: Ausschluss und Prekarisierung auf dem Arbeitsmarkt, der zu Armutsgefährdung, sozialer und räumlicher Isolation führt und dann schlussendlich in einem Gefühl des Ausschlusses aus der Gesellschaft resultiert, dem hier empirisch untersuchten Exklusionsempfinden. Dieser primär sozioökonomisch basierte Mechanismus greift aus Sicht der zweiten These relativ unspezifisch in unterschiedlichen Bevölkerungssegmenten, sodass er sozialstrukturell nicht klar zu verorten ist. Genau diese These der sozialstrukturellen Unspezifizität der sozialen Exklusion wollen wir auch in unserem Beitrag prüfen, indem wir die Strukturierung des Exklusionsempfindens nach Bildung, Alter, Staatsbürgerschaft und Sprachgruppe in der Schweiz fokussieren. An dieser Stelle soll kurz skizziert werden, warum überhaupt eine Exklusion nach diesen Merkmalen zu erwarten ist.

Es ist offensichtlich, dass die in der zeitdiagnostischen Konzeption der sozialen Exklusion angesprochenen Phänomene der Erwerbstätigkeit, Armut und Prekarität in Gegenwartsgesellschaften durch die Verfügung über Bildungsressourcen strukturiert sind, wobei Personen mit höherer Bildung und Qualifikation seltener erwerbslos und arm sind, dagegen häufiger höhere Einkommen beziehen. Dies zeigt sowohl die Forschung für den deutschsprachigen Raum als auch die internationale Forschung (Giesselmann und Vandecasteele 2018; Groh-Samberg 2014; Hümberlin und Fritschi 2016; Vandecasteele 2011; Oesch 2013; Preisner und Bertogg 2017). Schneickert et al. (2019) zeigen wiederum auf, dass der Exklusionsempfindung verwandte Phänomene, wie soziale Anerkennung und Geringschätzung, deutlich entlang der Bildungsdimension strukturiert sind. Daher werden wir die Zugehörigkeit zu Bildungsgruppen in unseren empirischen Analysen auch berücksichtigen.

Eine weitere mögliche Grundlage von sozialer Exklusion kann die Zugehörigkeit zu unterschiedlichen Altersgruppen darstellen. Folgt man hier dem Modell der Institutionalisierung des Lebenslaufs von Kohli (1997), das eine auf der Beteiligung am Erwerbsleben basierte Dreiteilung des Lebenslaufs skizziert: Vorbereitungs- (Kindheit und Jugend), Aktivitäts- (aktives Erwachsenenleben) und Ruhephase (Alter, Ruhestand), so können vor allem für die erste und dritte Phase mögliche Exklusionsrisiken festgestellt werden. In der Vorbereitungsphase, die durch Sozialisation und Ausbildung geprägt ist, sind jüngere Menschen im Alter der Adoleszenz und Postadoleszenz mit den Unsicherheiten der privaten und beruflichen Etablierung konfrontiert. Diese werden verstärkt durch die Tatsache, dass insbesondere jüngere Erwerbstätige stärker von Prekarisierungen betroffen sind als Erwerbstätige in mittleren Altersgruppen, die als Insider des Arbeitsmarktes in stärkerem Maße an Normalarbeitsverhältnissen partizipieren (Blossfeld und Mills 2005; Giesselmann 2009).

Spiegelbildlich dazu verhält sich die Situation älterer Bevölkerungsgruppen, die mit dem Ausscheiden aus dem Erwerbsleben, zunehmenden gesundheitlichen Problemen (insbesondere im Rahmen von Multimorbidität), dem rasanten technologischen Wandel und häufig auch der Ausdünnung sozialer Netzwerke konfrontiert sind (Chung et al. 2019; Lee und Cagle 2018). Die Nachberufsphase wird teilweise mit einer verminderten Möglichkeit der Teilhabe am sozialen Leben verbunden, da sozial relevante Rollen wegfallen (Dunkel et al. 2019, S. 74). Diese Gefühle der sozialen Exklusion verstärken sich, wenn das gesellschaftliche Altersbild negativ ist und Alter als Defizit im Leistungswettlauf sieht (Beyer et al. 2017). Eine aktuelle Exklusionsgefahr zeigt sich z. B. bei älteren Personen, welche keinen Zugang zum Internet haben oder neue technische Lösungen, wie z. B. den Videochat in Zeiten der durch COVID-19 verursachten physischen Distanzierungen, nicht nutzen können, um soziale Kontakte auf Distanz zu pflegen (Seifert et al. 2021). Je mehr sich das gesellschaftliche Leben auch im Internet abspielt – sei es über das Tätigen von Bankgeschäften via E‑Banking, das Stellen von Anträgen bei Ämtern und Behörden am Onlineschalter bis hin zum E‑Voting –, desto größer wird das Risiko sozialer Exklusion für Personen, die sich nicht daran beteiligen (Seifert et al. 2018).

Ein weiterer Faktor, der das Risiko sozialer Exklusion für Personen höheren Alters potenziell erhöht, ist der allgemeine Gesundheitszustand. So bringen beispielsweise Hajek und Koenig Stürze mit sozialer Isolation und Einsamkeit älterer Personen in Verbindung (Hajek und Koenig 2017). Kristensen et al. (2019) zeigen in einem Beitrag die Beziehung zwischen Multimorbidität, also dem gleichzeitigen Auftreten mehrerer Krankheitsleiden bei einer Person, und sozialer Exklusion auf. Ältere Personen können also auf der Grundlage unterschiedlicher sozialer Mechanismen (Soziale Netzwerke, Digitalisierung, Ruhestand, Gesundheitszustand) stärker von Exklusion betroffen sein.

Zusammenfassend betrachtet kann für die Dimension des Alters festgehalten werden, dass bisherige Studien nahelegen, dass verschiedene Prozesse der sozialen Exklusion altersspezifisch sind. Allerdings greifen hier ganz offensichtlich unterschiedliche Prozesse (Arbeitsmarkt, soziale Netzwerke, technologischer Wandel, Gesundheit), die sich nicht zwingend auf einen sozioökonomischen Mechanismus zurückführen lassen. Dabei kann angenommen werden, dass insbesondere für jüngere und ältere Menschen das Exklusionsrisiko erhöht ist, während dieses für Menschen in mittleren Altersgruppen auf einem niedrigeren Niveau liegt.

Eine weitere Personengruppe, die potenziell von sozialer Exklusion bedroht ist, sind Personen mit Migrationshintergrund, wobei wir hier auf Personen mit ausländischer Staatsangehörigkeit fokussieren (Saasa 2019). Im Jahr 2019 liegt der Anteil der Ausländer an der deutschen Gesamtbevölkerung bei rund 12,4 %, zudem weisen 13,6 % der Bevölkerung einen Migrationshintergrund auf (Bundeszentrale für politische Bildung 2020). Im Vergleich zu deutschen Staatsbürgern weisen diese Personen eine schlechtere Position in verschiedenen Lebensbereichen auf, wie z. B. dem Arbeits- und Wohnungsmarkt, dem Zugang zu Bildungseinrichtungen und sozialen Diensten. Ihre Beschäftigungsquote liegt niedriger als die von Deutschen, zudem sind sie stärker von Arbeitslosigkeit betroffen (Boeckh 2008, S. 370 f.; Rössel 2009, S. 219–222). Mit Blick auf die Bildungs- und Ausbildungsbeteiligung von Kindern und Jugendlichen ausländischer Herkunft kann festgestellt werden, dass diese weniger hohe Bildungsabschlüsse erwerben als deutsche Jugendliche und relativ häufig ohne allgemeinbildenden oder berufsbildenden Abschluss bleiben (Olczyk 2016).

Auch in der Schweiz kann ein Zusammenhang zwischen Staatsangehörigkeit und der individuellen Lebenslage festgestellt werden (Modetta 2019, S. 15). In der Schweiz lebten 2017 24,9 % ausländische Staatsangehörige und von den schweizerischen Staatsangehörigen haben zudem 12,5 % einen Migrationshintergrund (Modetta 2019, S. 11). Blickt man auf die finanzielle Situation dieser Personengruppen in der Schweiz, zeigen sich zunächst deutliche Unterschiede nach Nationalität und Staatsangehörigkeit, wobei insbesondere Personen aus ost- oder außereuropäischen Staaten über ungenügende finanzielle Reserven verfügen (Modetta 2019, S. 8). So kann festgestellt werden, dass ein deutlich höherer Anteil der süd-, ost- und außereuropäischen Staatsbürger erwerbslos ist als unter den Schweizern (Modetta 2019, S. 15). Vergleichbare Ergebnisse wie für Deutschland zeigen sich auch im Hinblick auf die Bildungs- und Ausbildungsbeteiligung. Auch hier verfügen ausländische Personen in der Schweiz, insbesondere Personen mit süd-, osteuropäischer und außereuropäischer Herkunft über deutlich schlechtere Bildungsabschlüsse als Schweizer. Gleiches gilt für den deutlich erhöhten Anteil von Jugendlichen, die das Bildungssystem ohne einen allgemeinbildenden oder beruflichen Bildungsabschluss verlassen (Becker und Beck 2012; Beck und Jäpel 2018).

Systematische Analysen der Lebenslage von Personen mit ausländischer Staatsangehörigkeit zeigen allerdings, dass die Unterschiede zu inländischen Staatsangehörigen überwiegend nicht auf migrationsspezifische Ursachen zurückzuführen sind, sondern primär durch die soziale Herkunft bedingt sind. Darüber hinaus spielen als wichtige Ursachen die Kenntnisse der jeweiligen Landessprache und Prozesse der Diskriminierung eine Rolle (Esser 2006; Nauck 2016; Kempert 2016). Während es im Bereich der Allgemeinbildung empirisch nur wenige Hinweise auf die Diskriminierung von ausländischen Jugendlichen gibt, liegen für den Übergang zur beruflichen Ausbildung und auf dem Arbeitsmarkt sowohl für Deutschland als auch die Schweiz klare Hinweise für eine solche Diskriminierung vor (Diehl und Fick 2016; Fibbi et al. 2006; Koopmans et al. 2019; Auer et al. 2015; Zschirnt und Ruedin 2016; Heath et al. 2008). Dies gilt in gleichem Maße für den Wohnungsmarkt (Auspurg et al. 2019). Insofern existiert für Personen mit ausländischer Staatsangehörigkeit in Prozessen der Diskriminierung eine mögliche Ursache von spezifischen Exklusionsprozessen, die im zeitdiagnostischen Modell nicht enthalten ist. Dies spiegelt sich auch in der von Personen mit Migrationshintergrund subjektiv wahrgenommenen Diskriminierung wider (Diehl et al. 2021). Für unsere Fragestellung ist von zentraler Bedeutung, dass wahrgenommene Diskriminierung zu einer verringerten Identifikation mit der Aufnahmegesellschaft führt (Diehl et al. 2021) – ausländische Staatsbürger und Personen mit Migrationshintergrund fühlen sich also in dieser Situation nicht als Teil der Aufnahmegesellschaft, sondern, in der hier gewählten Begrifflichkeit, exkludiert.

Werden für Deutschland schließlich geographische Differenzierungen häufig entlang der Unterscheidung von West- und Ostdeutschland untersucht (s. Rössel 2009; Schneickert et al. 2019), so liegt es für die Schweiz nahe, den Fokus auf die unterschiedlichen Sprachgruppen zu richten, die in geographisch klar abgegrenzten Räumen leben (Linder und Steffen 2006). Hier sind aufgrund ihrer Minderheitenposition insbesondere die französisch- und die italienischsprachigen Schweizer vom Risiko eines erhöhten Exklusionsempfindens betroffen (Kriesi et al. 1996). Im Gegensatz zu anderen multilingualen Ländern, wie Belgien, Kanada und Spanien, war der sprachregionale Gegensatz in der Schweiz zwar kaum Grundlage von größeren sozialen und politischen Konflikten (Pinard 2020), dennoch wird diese Konfliktlinie auch in der politischen Öffentlichkeit unter den alltagssprachlichen Begriffen des „Röstigrabens“ und des „Polentagrabens“ wahrgenommen.^6^ Diese Wahrnehmung ist bei den sprachlichen Minderheiten stärker verankert, werden diese doch in politischen Diskussionen und Abstimmungen durch die Mehrheit der Deutschschweizer immer wieder majorisiert (Kriesi et al. 1996; Linder und Steffen 2006). In zentralen politischen Fragen ist es damit die deutschsprachige Mehrheit, die die politische Ausrichtung der schweizerischen Gesellschaft dominiert und damit zu einem Gefühl des Ausgeschlossenseins bei den sprachlichen Minderheiten führen kann.

Auf der Basis der bisherigen Ausführungen sollen in unserer empirischen Untersuchung des Exklusionsempfindens vier Gesichtspunkte im Zentrum unserer Fragestellung stehen: Erstens soll, entsprechend der ersten These des zeitdiagnostischen Konzepts der Exklusion, die Verbreitung des Exklusionsempfindens in der Bevölkerung geprüft werden. Die Annahme, dass es sich hier um die zentrale Spaltungslinie der Gesellschaft handelt, legt eine weite Verbreitung von Exklusionsempfindungen nahe, unsere Diskussion von Prozessen der sozialen Exklusion (Abschn. 2.1) spricht eher dafür, dass sich lediglich kleine Bevölkerungsgruppen exkludiert fühlen. Zweitens soll die zweite These der sozialstrukturellen Unspezifizität des Exklusionsempfindens empirisch getestet werden. Dazu werden wir die Relevanz von Bildung, Alter, Staatsbürgerschaft und Sprachgruppe für das Exklusionsempfinden prüfen. Wir vermuten hier, im Gegensatz zum zeitdiagnostischen Konzept, eine deutliche Strukturierung des Exklusionsempfindens entlang dieser sozialstrukturellen Kategorien. Drittens werden im zeitdiagnostischen Modell als dritte These insbesondere sozioökonomische Marginalisierung (Erwerbstätigkeit, Armut) und soziale Isolation als zentrale Triebkräfte von sozialer Exklusion behandelt. Diese sollen in ihrer Relevanz für das Exklusionsempfinden empirisch untersucht werden. Schließlich soll viertens die subjektive Wahrnehmung der persönlichen Lage, sowohl im Hinblick auf sozioökonomische Bedingungen als auch im Hinblick auf soziale Isolation, entsprechend der vierten These als vermittelnde Variable betrachtet werden, so wie dies auch im Modell von Bude und Lantermann (2006) vorgeschlagen wurde.

## Daten und Methode

Zur Beantwortung der Forschungsfragen greifen wir auf eine repräsentative Bevölkerungsbefragung zum Thema „Digitaler Alltag in der Schweiz“ zurück. Adressgrundlage für die Befragung bildet der Stichprobenrahmen des Bundesamts für Statistik, aus dem eine disproportionale, nach Alter und Sprachregion geschichtete Zufallsstichprobe gezogen wurde. Die Mixed-Mode-Befragungsmethode umfasste computergestützte Onlineinterviews (CAWI) und computergestützte Telefoninterviews (CATI), die in den Schweizer Landessprachen Deutsch, Französisch und Italienisch durchgeführt wurden. Außerdem wurden postalische und personalisierte Einladungen und zwei Erinnerungsschreiben verschickt. Die Interviewdauer betrug über die verschiedenen Befragungsmethoden im Mittel 41,8 min. Zwischen dem 29.10.2019 und dem 17.12.2019 wurden insgesamt 1659 Interviews (CAWI und CATI) mit in der Schweiz wohnhaften Personen ab 18 Jahren realisiert. Damit wurde eine Nettoausschöpfungsquote von 30,5 % erreicht. Die folgenden Analysen haben wir unter Ausschluss der Fälle mit fehlenden Werten durchgeführt. Im Process-Modul von Hayes (2013), das wir für die Durchführung von Mediationsanalysen verwendet haben, kann keine multiple Imputation vorgenommen werden, weshalb wir uns für den Ausschluss der Fälle mit fehlenden Werten entschieden haben. Um die Robustheit unserer Ergebnisse zu prüfen, haben wir in SPSS eine MCMC-Imputation der fehlenden Werte vorgenommen (jeweils 20 geschätzte Werte) und die statistischen Modelle auch mit den imputierten Daten berechnet. Die Resultate zeigen nur geringfügige Unterschiede, sodass wir hier die Ergebnisse für die Analysen unter Ausschluss der Fälle mit fehlenden Werten präsentieren.

Auf der Grundlage des Datensatzes können wir die zentralen verwendeten Variablen operationalisieren. Zunächst wurde die abhängige Variable *Exklusionsempfinden* gebildet. Dafür wurde im Anschluss an die Skala von Bude und Lantermann (2006) die Zustimmung der Befragten zu verschiedenen Aussagen nach dem wahrgenommenen Gefühl der sozialen Exklusion („Ich habe Angst, den Anschluss zu verpassen“, „Ich habe das Gefühl, andere Menschen haben mich abgeschrieben“, „Ich werde ausgegrenzt“, „Ich habe das Gefühl, gar nicht richtig zur Gesellschaft zu gehören“, „Ich habe das Gefühl, im Grunde gesellschaftlich überflüssig zu sein“) in einer Variable (es wurde der Mittelwert über alle Einzelitems gebildet) zusammengefasst (Cronbachs Alpha = 0,87). Die Einzelitems wurden auf einer 5‑stufigen Skala mit Antwortmöglichkeiten von „trifft voll und ganz zu“ bis „trifft überhaupt nicht zu“ erfasst. Da die Gesamtskala eine linkssteile Verteilung aufweist, wurde sie für die multivariaten statistischen Analysen logarithmiert.

Um die vermuteten Zusammenhänge des Exklusionsempfindens mit sozialen Gruppenzugehörigkeiten zu testen (These 2), werden folgende soziodemografische Variablen berücksichtigt: *Bildung* (in drei Kategorien aufgeteilt: Obligatorischer Abschluss, Abschluss der Sekundarstufe, Abschluss der Tertiärstufe), *Alter* (in vier Kategorien aufgeteilt), *Staatsangehörigkeit*, die *Schweizer Sprachregion* sowie als Kontrollvariable das *Geschlecht*. Wir haben die älteren Bevölkerungsgruppen in zwei Segmente aufgeteilt, da sich in verschiedenen Studien deutliche Unterschiede zwischen älteren Menschen im frühen Ruhestandsalter und den Hochbetagten gezeigt haben (Baltes und Smith 2003).

Über die soziodemografischen Variablen hinaus berücksichtigen wir in unseren statistischen Analysen, die im zeitdiagnostischen Modell angesprochenen objektiven Dimensionen der Lebenslage, die zu einer Marginalisierung beitragen können (These 3): Erwerbstätigkeit^7^, Pro-Kopf-Einkommen, soziale Netzwerke (Partnerschaft, Kontakt zu den eigenen Kindern, Kontakt zu Familienangehörigen als dichotome Variablen wurden zu einem Index aufsummiert) sowie das regelmäßige Treffen enger Freunde (dichotom kodiert). Das *Pro-Kopf-Einkommen* wurde nach der neuen OECD-Skala über die Haushaltsgröße und das Haushaltseinkommen ermittelt.

Zusätzlich zu den soziodemografischen Variablen und den objektiven Informationen zur Lebenslage haben wir auch die subjektive Einschätzung verschiedener Dimensionen der Lebenslage erfasst. Damit sollte die auch im Modell von Bude und Lantermann (2006) angesprochene psychologische Verarbeitung der eigenen Lebenslage erfasst werden (These 4). Die Variable *Einsamkeit *basiert auf der Zustimmung der Befragten zu drei Aussagen (5-stufige Likert-Skala mit Antwortmöglichkeiten von „nie“ bis „sehr oft“) zur wahrgenommenen sozialen Isolation („Wie häufig haben Sie das Gefühl … – dass Ihnen die Gesellschaft anderer fehlt?“, „Wie häufig haben Sie das Gefühl … – außen vor/nicht dabei zu sein?“, „Wie häufig haben Sie das Gefühl … – dass Sie sozial isoliert sind?“), die zu einer Skala zusammengefasst wurden (Cronbachs Alpha = 0,83). Darüber hinaus wurde die Zufriedenheit mit der finanziellen Situation, der Wohnsituation und der eigenen Gesundheit mit je einem Item erfasst (jeweils eine 5‑stufige Antwortmöglichkeit mit den Antwortmöglichkeiten von „sehr schlecht“ bis „sehr gut“). Ferner konnte auch eine Skala zur Einschätzung der Digitalisierung berücksichtigt werden. Die Variable *Digitale Exklusion* basiert auf der Zustimmung der Befragten zu drei Aussagen (5-stufige Likert-Skala mit Antwortmöglichkeiten von „trifft überhaupt nicht zu“ bis „trifft voll zu“) zum Thema digitale Exklusion („Bitte geben Sie an, wie sehr Sie den folgenden Aussagen zustimmen.“ – „Ich habe das Gefühl, ausgegrenzt zu sein, wenn ich die neusten technischen Geräte nicht beherrsche“, „Man muss heute digitale Dienstleistungen nutzen, um mitreden zu können“, „Es sollten weiterhin Vor-Ort-Angebote von Dienstleistungen erhalten bleiben (z. B. klassische Poststelle vor Ort)“), die zu einer Skala zusammengefasst wurden (Cronbachs Alpha = 0,71). Die Mittelwerte und Streuungsmaße der verwendeten Variablen finden sich in Tab. 1 im Online-Anhang.

## Empirische Ergebnisse

Die empirische Untersuchung führen wir in zwei Schritten durch. Im ersten Schritt werfen wir unser Augenmerk auf das Ausmaß von subjektiv wahrgenommener Exklusion in der Bevölkerung und seine Verteilung, um die erste These zu untersuchen. Da es sich hier nur um eine einfache deskriptive Fragestellung handelt, reicht die Betrachtung von Mittelwerten und relativen Häufigkeiten. Im zweiten Schritt führen wir OLS-Regressionsanalysen durch, welche die Kovariation des Exklusionsempfindens untersuchen. Wir folgen dabei einer schrittweisen Vorgehensweise: im ersten Regressionsmodell werden nur die soziodemografischen Unterschiede entsprechend der zweiten These berücksichtigt (Bildung, Alter, Staatsbürgerschaft, Sprachregion). Im zweiten Schritt werden die spezifischen ökonomischen und sozialen Ressourcen, die relevant für den im zeitdiagnostischen Modell beschriebenen Exklusionsprozess sind, also Erwerbstätigkeit, Einkommen und soziale Kontakte, mit in das Modell aufgenommen (These 3), während im dritten Modell die tatsächliche Wahrnehmung von Problemen in einzelnen Lebenslagen (Finanzen, Wohnung, soziale Isolation, Digitalisierung, Gesundheit) entsprechend dem von Bude und Lantermann (2006) vermuteten psychologischen Verarbeitungsmodell berücksichtigt wird (These 4). Wir haben darüber hinaus geprüft, ob die Unterschiede zwischen den Gruppen in Modell 1 durch die in Modell 2 berücksichtigten ökonomischen und sozialen Ressourcen sowie die in Modell 3 analysierten Wahrnehmungen dieser Ressourcenausstattung erklärt werden können. Dazu haben wir Mediationsanalysen durchgeführt, deren signifikante Ergebnisse in Tab. A2 im Online-Anhang aufgeführt wurden. In einer Mediationsanalyse wird betrachtet, ob der Zusammenhang zwischen einer Variable X und einer Variable Y über eine dazwischenliegende Variable M vermittelt wird. Den Effekt von X auf Y, der über M vermittelt wird, bezeichnet man als indirekten Effekt. Es könnte sich hier um den Effekt der Bildung (X) auf das Exklusionsempfinden (Y) handeln. Dieser könnte über das Einkommen (M) vermittelt werden. Höhere Bildung (X) hat einen positiven Effekt auf das Einkommen (M) und dieses wiederum einen Einfluss auf das Exklusionsempfinden (Y). In diesem Fall gäbe es also einen über das Einkommen vermittelten indirekten Effekt der Bildung auf das Exklusionsempfinden. Es gibt verschiedene Tests, um zu prüfen, ob die indirekten Effekte statistisch signifikant sind oder nicht. In Simulationsstudien konnte gezeigt werden, dass insbesondere nichtparametrische Verfahren (Bootstrapping) sich für die Schätzung des Signifikanztests besonders gut eignen. Wir greifen hier auf den von Hayes (2013) entwickelten nichtparametrischen Schätzer zurück, um das Konfidenzintervall der indirekten Effekte zu schätzen. Da kein klar ausdifferenziertes theoretisches Modell für die Erklärung des Exklusionsempfindens vorliegt, sind wir explorativ vorgegangen und haben alle möglichen Mediationsbeziehungen auf der Basis des von Hayes (2013) nichtparametrischen Verfahrens überprüft. Diese greifen wir in der Interpretation der Resultate auszugsweise auf.

Für die Darstellung der Ergebnisse in Tab. 1 haben wir das Exklusionsempfinden entlang seiner Spanne (1–5) in drei gleich breite Bereiche aufgeteilt. Die Skala des Exklusionsempfindens weist eine ausgesprochen schiefe Verteilung auf. Lediglich 2,9 % der Befragten erreichen Werte im oberen Drittel, fühlen sich also subjektiv eindeutig exkludiert. Dagegen finden sich bei den Werten im unteren Drittel rund 80 % der Befragten. Damit kann entsprechend der ersten These festgehalten werden, dass nur ein minimaler Bevölkerungsanteil sich tatsächlich als subjektiv exkludiert wahrnimmt. Allerdings befinden sich fast 17 % der Befragten im mittleren Drittel des Wertebereichs: Diese sehen sich zwar nicht als exkludiert an, aber offenbar auch nicht als vollständig inkludiert. Auch wenn dieser Wert nicht für die Zeitdiagnose der sozialen Exklusion spricht, gibt es also durchaus eine nicht unerhebliche Gruppe von Menschen, die Zweifel an ihrer Vollinklusion in die Gesellschaft aufweisen. Betrachtet man die weiteren Ergebnisse in Tab. 1 als eine erste deskriptive Annäherung der gruppenspezifischen Unterschiede im Anschluss an These 2, so zeigen sich deutliche Hinweise auf die sozialstrukturelle Verteilung des Exklusionsempfindens. Wir haben hier alle Gruppierungsvariablen berücksichtigt, die auch im ersten Regressionsmodell analysiert werden. Es zeigen sich die erwarteten Zusammenhänge zwischen der Bildung einerseits und dem Exklusionsgefühl andererseits, wobei sich Personen mit geringerer Bildung häufiger als exkludiert empfinden. Dagegen zeigt sich beim Alter ein u‑förmiger Zusammenhang, wobei sich bei den jüngeren und den älteren Befragten ein etwas größerer Anteil als stärker exkludiert wahrnimmt, wobei entgegen dem Modell des dreigeteilten Lebenslaufs für ältere Menschen tatsächlich erst im hochbetagten Alter die Wahrscheinlichkeit für das Exklusionsempfinden ansteigt und nicht schon mit dem Übergang in den Ruhestand. Darüber hinaus wird deutlich, dass auch Personen ohne Schweizer Staatsangehörigkeit sowie Personen aus den beiden kleineren Sprachgruppen der französisch- und italienischsprachigen Schweiz sich häufiger als exkludiert wahrnehmen. Es ist auffällig, dass beim Exklusionsempfinden kaum geschlechtsspezifische Unterschiede existieren, Frauen und Männer fühlen sich in gleichem Maße in die Gesellschaft inkludiert. Schon diese deskriptiven Resultate deuten darauf hin, dass soziale Exklusion ein komplexeres Phänomen ist als es in der zeitdiagnostischen Verwendung des Konzepts angenommen wird. Nicht allein entlang sozioökonomischer Differenzierungslinien zeigt sich ein unterschiedliches Ausmaß des Exklusionsempfindens, sondern auch nach Alter, Staatsbürgerschaft und Sprachregion.

**Table 1:** 

		Gering	Mittel	Hoch	Total
*Geschlecht*	Weiblich	672 80,9 %	138 16,6 %	21 2,5 %	831 100 %
	Männlich	613 79,7 %	130 16,9 %	26 3,4 %	769 100 %
	Total	1285 80,3 %	268 16,8 %	47 2,9 %	1600 100 %
*Alter*	18–30 Jahre	172 72,6 %	57 24,1 %	8 3,4 %	237 100 %
	31–60 Jahre	680 82,2 %	134 16,2 %	13 1,6 %	827 100 %
	61–75 Jahre	313 83,9 %	45 12,1 %	15 4,0 %	373 100 %
	76–98 Jahre	120 73,6 %	32 19,6 %	11 6,8 %	163 100 %
	Total	1285 80,3 %	268 16,8 %	47 2,4 %	1600 100 %
*Staatsangehörigkeit*	Schweiz	1088 81,7 %	206 15,5 %	37 2,3 %	1331 100 %
	Andere	197 73,2 %	62 23,1 %	10 3,7 %	269 100 %
	Total	1285 100,0 %	268 100,0 %	47 100,0 %	1600 100 %
*Sprachregion*	Deutschschweiz	772 85,2 %	123 13,6 %	11 1,2 %	906 100 %
	Romandie	306 73,4 %	94 22,6 %	17 4,1 %	417 100 %
	Italienische Schweiz	203 74,4 %	51 18,7 %	19 7,0 %	273 100 %
	Total	1281 80,3 %	268 16,8 %	47 3,0 %	1596 100 %
*Bildung*	Obligatorische Schule	115 71,4 %	38 23,6 %	8 5,0 %	161 100 %
	Sekundarstufe II	577 79,3 %	127 17,5 %	24 3,3 %	728 100 %
	Tertiärstufe	556 83,5 %	98 14,7 %	12 1,8 %	666 100 %
	Total	1248 80,3 %	263 16,9 %	44 2,8 %	1555 100 %
–	Total	1269 80,4 %	265 16,8 %	45 2,9 %	1579 100 %

Aufteilung Exklusionsempfinden: „gering“: 1–2,30, „mittel“: 2,31–3,60, „hoch“: 3,61–5,00

Die im vorhergehenden Abschnitt dargestellten deskriptiven Resultate werden im zweiten Schritt im Rahmen einer multivariaten Regressionsanalyse umfassender geprüft.

Wir beginnen die Interpretation der Resultate mit Modell 1 in Tab. 2. Diese bestätigen die Ergebnisse der deskriptiven Auswertung in Tab. 1 und sprechen recht eindeutig gegen die zweite zentrale These des zeitdiagnostischen Exklusionskonzepts, die Idee der sozialstrukturellen Unspezifizität. Für die Bildungsvariable zeigt sich, dass Personen mit einem Abschluss der Tertiärstufe sich signifikant seltener als exkludiert wahrnehmen als Personen mit einem obligatorischen Schulabschluss. Personen mit einem Sekundarschulabschluss unterscheiden sich dagegen nicht signifikant von denjenigen mit einem obligatorischen Schulabschluss. Betrachtet man das Alter, so sind es vor allem die mittleren Altersgruppen, die sich weniger stark exkludiert fühlen. Auch Personen mit Schweizer Staatsbürgerschaft nehmen sich als weniger exkludiert wahr. Auffällig sind die klaren Unterschiede zwischen den Sprachregionen in der Schweiz. Hier zeigt sich sehr deutlich, dass die Sprecher der beiden größeren Minderheitensprachen in der Schweiz sich stärker exkludiert fühlen als die Deutschschweizer. Damit spricht Modell 1 deutlich gegen die zweite These des zeitdiagnostischen Exklusionskonzepts, die Idee der sozialstrukturellen Unspezifizität von sozialer Exklusion. Ferner wird auch deutlich, dass soziale Exklusion in Abhängigkeit von unterschiedlichen sozialstrukturellen Dimensionen variiert.

**Table 2:** 

	Modell I			Modell II			Modell III		
	Unstandardisierte Koeffizienten	Standardisierte Koeffizienten	t‑Wert	Unstandardisierte Koeffizienten	Standardisierte Koeffizienten	t‑Wert	Unstandardisierte Koeffizienten	Standardisierte Koeffizienten	t‑Wert
Konstante	0,503***	–	10,440	0,629***	–	11,287	0,437***	–	3,893
Geschlecht männlich (Referenzkategorie: weiblich)	0,013	0,016	0,548	0,035	0,044	1,530	0,032	0,040	1,654
Referenzkategorie Altersgruppe 31–60									
Altersgruppe 18–30	0,136***	0,114***	3,857	0,083*	0,069*	2,194	0,062	0,052	1,892
Altersgruppe 61–75	−0,010	−0,010	−0,384	−0,047	−0,050	−1,421	−0,01	−0,012	−0,382
Altersgruppe 76–98	0,092*	0,069*	2,288	0,015	0,011	0,323	0,030	0,022	0,747
Schweizer Staatsangehörigkeit (Referenzkategorie: andere Staatsangehörigkeit)	−0,123***	−0,107***	−3,678	−0,115***	−0,100***	−3,530	−0,025	−0,022	−0,895
Französische Schweiz (Referenzkategorie: Deutschschweiz)	0,187***	0,201***	6,839	0,193***	0,208***	7,161	0,097***	0,105***	4,078
Italienische Schweiz (Referenzkategorie: Deutschschweiz)	0,118***	0,106***	3,595	0,110**	0,099**	3,362	0,109***	0,098***	3,883
Sekundarstufe II (Referenzkategorie: Obligatorische Schule)	−0,012	−0,015	−0,295	0,027	0,034	0,659	0,013	0,016	0,370
Tertiärstufe (Referenzkategorie: Obligatorische Schule)	−0,109**	−0,135**	−2,617	−0,043	−0,053	−1,011	−0,026	−0,032	−0,716
Pro-Kopf-Einkommen	–	–	–	−2,346E^−5^***	−0,118***	−3,826	−7,040E^−6^	−0,035	−1,285
Erwerbstätigkeit	–	–	–	−0,0083**	−0,100**	−2,650	−0,058*	−0,069*	−2,142
Soziales Netzwerk	–	–	–	−0,236***	−0,122***	−4,040	−0,123*	−0,063*	−2,434
Regelmäßiges Treffen enger Freunde	–	–	–	−0,067***	−0,148***	−5,273	−0,023*	−0,050*	−2,028
Zufriedenheit mit finanz. Situation	–	–	–	–	–	–	−0,073***	−0,162***	−5,721
Zufriedenheit mit Wohnsituation	–	–	–	–	–	–	−0,011	−0,019	−0,753
Selbsteingeschätzter Gesundheitszustand	–	–	–	–	–	–	−0,030*	−0,054*	−2,119
Einsamkeit	–	–	–	–	–	–	0,220***	0,424***	16,235
Digitale Exklusion	–	–	–	–	–	–	0,038***	0,092***	3,766
R^2^ (korrigiert)	0,089 (0,082)	–	–	0,139 (0,128)	–	–	0,379 (0,369)	–	–
*N*	1158	–	–	1158	–	–	1158	–	–

Signifikanzniveau: * *p* ≤ 0,05, ** *p* ≤ 0,01, *** *p* ≤ 0,001

Wir betrachten nun im Anschluss an These 3 im nächsten Schritt in Modell 2 die Effekte von spezifischen Ressourcen auf das Exklusionsempfinden. Entsprechend der Fokussierung des zeitdiagnostischen Exklusionskonzepts auf sozioökonomische Ressourcen und soziale Isolation wurden hier das Einkommen, die Erwerbstätigkeit und zwei Indikatoren für die sozialen Kontakte aufgenommen. Es wird deutlich, dass Erwerbstätigkeit und ein höheres Einkommen die wahrgenommene Exklusion reduzieren. Vergleicht man Modell 1 und Modell 2, so zeigt sich, dass der Effekt der Bildung verschwindet, sobald das Einkommen berücksichtigt wird. Dies kann durch eine Mediationsanalyse bestätigt werden (Tab. A2 im Online-Anhang).^8^ Die beiden Ergebnisse zusammen sprechen allerdings deutlich für die Annahme, dass sozioökonomisch benachteiligte Gruppen ein größeres Exklusionsempfinden aufweisen. Alle Indikatoren für die sozialen Kontakte sind dagegen negativ mit einer Exklusionswahrnehmung korreliert. Die sozioökonomische Lage und die soziale Isolation tragen offensichtlich unabhängig voneinander zur Exklusionswahrnehmung bei. Betrachtet man die Effektgrößen der weiteren Variablen aus Modell 1, so zeigt sich in Modell 2 lediglich, dass die Alterseffekte kleiner werden. Diese werden sowohl durch das Einkommen als auch durch die sozialen Kontakte vermittelt (Tab. 2 im Online-Anhang). Dagegen verändern sich die Effektgrößen der Staatsbürgerschaft und der Sprachregionen in Modell 2 im Vergleich zu Modell 1 kaum.

In Modell 3 haben wir in fünf Dimensionen auch die Wahrnehmung von Ressourcen und Problemlagen berücksichtigt, um These 4 zu untersuchen. Im Anschluss an die Diskussion über räumliche Segregation haben wir die Zufriedenheit mit der Wohnsituation ergänzend aufgenommen. Diese kovariiert allerdings nicht signifikant mit der wahrgenommenen Exklusion. Dagegen weist die Zufriedenheit mit der finanziellen Situation einen erheblichen Einfluss auf die wahrgenommene Exklusion auf. Der statistische Effekt für das Einkommen verschwindet bei Berücksichtigung der finanziellen Zufriedenheit, wird also durch diese vermittelt (Tab. A2 im Online-Anhang). Als dritte Dimension der Einschätzung der persönlichen Lage wurde das Gefühl der Einsamkeit als Indikator der wahrgenommenen sozialen Isolation berücksichtigt. Diese Variable kovariiert sehr stark mit dem Exklusionsempfinden. Die Effekte der objektiven Indikatoren der sozialen Isolation (soziales Netzwerk, regelmäßiges Treffen enger Freunde) verringern sich unter Berücksichtigung der Einsamkeit sehr deutlich, werden also durch diese vermittelt (Tab. A2 im Online-Anhang). Schließlich wurde über diese drei Dimensionen des zeitdiagnostischen Konzepts hinausgehend auch die subjektive Wahrnehmung des Gesundheitszustands berücksichtigt. Diese korreliert signifikant, aber nicht stark mit der wahrgenommenen Exklusion. Zudem wurde auch die Einschätzung der Digitalisierung berücksichtigt. Auch hier kann festgestellt werden, dass Menschen, die mit der Digitalisierung der Gesellschaft hadern, sich als stärker exkludiert empfinden. Insgesamt spricht die Untersuchung dieser fünf Indikatoren der Einschätzung der persönlichen Lage stark dafür, dass die Wahrnehmung von Exklusion entsprechend These 4 deutlich durch subjektive Verarbeitungsprozesse der objektiven Ressourcenausstattung geprägt ist. Diese vermitteln auch die größere Exklusionswahrnehmung der Personen, die keine Schweizer Staatsbürger sind. Der Effekt dieser Variable ist im dritten Modell nicht mehr statistisch signifikant und wird über die Wahrnehmung der finanziellen Situation und der wahrgenommenen Einsamkeit vermittelt (Tab. A2 im Online-Anhang). Dagegen verringern sich die Effektgrößen für die Sprachregionen nur geringfügig, diese können also weder durch die objektive Lage noch deren subjektive Wahrnehmung erklärt werden.

## Zusammenfassung und Diskussion

Das Konzept der sozialen Exklusion hat sich in den Sozialwissenschaften in unterschiedlichen Verwendungsweisen etabliert. In unserem Beitrag haben wir als eine gesellschaftstheoretische Variante die zeitdiagnostische Konzeption mit ihren zentralen Thesen aufgegriffen. Aufgrund der zentralen Bedeutung von sozialer Zugehörigkeit als menschlichem Grundbedürfnis haben wir einen von Bude und Lantermann entwickelten Index zur Messung des Exklusionsempfindens in das Zentrum der empirischen Studie gestellt, um vier Thesen dieser zeitdiagnostischen Konzeption empirisch zu untersuchen.

Wir haben erstens geprüft, ob sich tatsächlich ein erheblicher Anteil von Menschen exkludiert fühlt, sodass dieses Konzept tatsächlich die zentrale Spaltungslinie von Gegenwartsgesellschaften erfassen kann, wie dies im zeitdiagnostischen Exklusionskonzept als erste zentrale These behauptet wird. Wir konnten in unserer Literaturdiskussion zur zeitdiagnostischen Verwendung des Exklusionskonzepts aufzeigen, dass der Anteil von Menschen, die in verschiedenen Dimensionen gleichzeitig exkludiert sind (Armut, prekäre Arbeitsverhältnisse, soziale Isolation, residentielle Segregation) in einem einstelligen Prozentbereich zu vermuten ist. Entsprechend zeigt sich auch in unseren Daten, dass sich lediglich 2,9 % der Befragten als exkludiert empfinden. Allerdings zeigt sich auch, dass fast 17 % der Befragten zumindest Zweifel an ihrer Teilhabe in der Gesellschaft haben. Diese haben im Durchschnitt einen mittleren Wert im Hinblick auf ihr Exklusionsempfinden angegeben. Diese empirischen Resultate können die erste zentrale These des zeitdiagnostischen Exklusionskonzepts nur begrenzt unterstützen.

Zweitens haben wir die zweite These des zeitdiagnostischen Exklusionskonzepts untersucht, die sozialstrukturelle Unspezifizität von Exklusionswahrnehmung. Unsere Analysen ergaben im Gegensatz zu dieser These eine deutliche sozialstrukturelle Verortung von Exklusionswahrnehmung. Diese war erstens entlang von Bildungsunterschieden geprägt. Zweitens ergaben die Analysen auch, dass das Alter einen u‑förmigen Zusammenhang mit der Wahrnehmung von Exklusion aufweist. Sowohl eher jüngere als auch eher hochbetagte Menschen fühlen sich stärker exkludiert als Menschen in mittleren Altersgruppen. Ferner konnte in den Analysen drittens aufgezeigt werden, dass Menschen ohne Schweizer Staatsbürgerschaft sich in stärkerem Maße exkludiert fühlen. Schließlich konnte viertens auch festgestellt werden, dass die beiden großen sprachlichen Minderheiten in der Schweiz, die französisch- und die italienischsprachige Bevölkerung, sich in stärkerem Maße als ausgeschlossen wahrnehmen. Das Exklusionsempfinden ist also erstens als sozialstrukturell geprägt zu betrachten und zweitens durch unterschiedliche Dimensionen der Sozialstruktur geprägt und nicht allein von einer klassischen vertikalen Perspektive.

Drittens haben wir in unserer Studie die These untersucht, ob die sozioökonomische Lage und die soziale Isolation von Personen, als zentrale Elemente des Exklusionsprozesses im zeitdiagnostischen Modell, mit der Exklusionswahrnehmung kovariieren. Dies konnte deutlich bestätigt werden. Dies spricht dafür, dass der im zeitdiagnostischen Modell angesprochene Mechanismus der sozialen Exklusion tatsächlich relevant ist. Allerdings konnten die Unterschiede in der Exklusionswahrnehmung zwischen Schweizer Staatsbürgern und Ausländern sowie zwischen den Schweizer Sprachregionen auf der Grundlage von objektiven Divergenzen der sozioökonomischen Lage und der sozialen Isolation nicht erklärt werden.

Viertens haben wir im Anschluss an das Modell von Bude und Lantermann (2006) auch die These geprüft, ob die subjektive Wahrnehmung der eigenen Lage für das Exklusionsempfinden eine entscheidende Rolle spielt. Hier wurde deutlich, dass insbesondere die wahrgenommene Einsamkeit, aber auch die Einschätzung der eigenen finanziellen Situation von besonderer Bedeutung sind. Die Rolle der objektiven sozioökonomischen Lage und der objektiven sozialen Kontakte wurde fast vollständig über deren subjektive Wahrnehmung statistisch vermittelt. Darüber hinaus zeigten sich auch gewisse Kovariationen der Einschätzung der eigenen Gesundheit und der Digitalisierung mit dem Exklusionsempfinden.

Die vorgestellten Argumente auf der Basis der Literaturdiskussion und der empirischen Analyse sprechen dafür, dass die zeitdiagnostische Verwendung des Exklusionsbegriffs nur begrenztes diagnostisches Potenzial aufweist. Die von Exklusion im Sinne dieses Konzepts betroffenen Personengruppen sind so klein, dass in keinem ernsthaften Sinne von einer zentralen Spaltung gegenwärtiger Gesellschaften gesprochen werden kann. Allerdings muss berücksichtigt werden, dass ein nicht unerheblicher Anteil der Bevölkerung sich nicht als vollständig inkludiert wahrnimmt. Auch kann festgestellt werden, dass sich als exkludiert empfindende Gruppen sozialstrukturell recht klar bestimmen lassen. Insofern kann auch der These, dass sich Exklusion sozialstrukturell nicht mehr verorten ließe, klar widersprochen werden. Schließlich zeigt sich aber auch, dass der primäre Fokus der zeitdiagnostischen Verwendung des Konzepts auf sozioökonomischen Strukturwandel und anschließende Prozesse der sozialen und räumlichen Isolation durchaus eine Teilerklärung von Exklusionserleben sein kann. Wahrgenommene soziale Exklusion taucht in unterschiedlichen Gruppen verstärkt auf, bei jüngeren und älteren Menschen, bei Menschen mit ausländischer Staatsangehörigkeit und interessanterweise auch klar ausgeprägt bei den sprachlichen Minderheiten in der Schweiz. Es ist offensichtlich, dass die Erklärung von Exklusion in diesen verschiedenen Gruppen auf unterschiedliche soziale Mechanismen fokussieren muss und nicht ausschließlich auf die im zeitdiagnostischen Modell insinuierten Mechanismen. So scheinen Ausländer ihre Lage subjektiv anders wahrzunehmen als Staatsbürger, während die Unterschiede zwischen den Sprachregionen sich über die hier betrachteten Mechanismen praktisch nicht erklären lassen. Wie in Abschn. 3.2 erläutert, vermuten wir hier vor allem einen Effekt der gesellschaftlichen und politischen Majorisierung durch die Mehrheit der Deutschschweizer. Aufgrund der großen Bedeutung, die Prozesse der sozialen Exklusion für die betroffenen Personen haben, ist es von zentraler Bedeutung, präzise Erklärungen für diese Prozesse zu formulieren. Ein One-fits-all-Konzept, wie das der zeitdiagnostischen Deutung von sozialer Exklusion, hilft hier nicht weiter.

Zum Abschluss sollen noch drei Limitationen unserer Studie hervorgehoben werden. Erstens muss berücksichtigt werden, dass die empirischen Daten nicht für die Untersuchung des zeitdiagnostischen Konzepts von sozialer Exklusion erhoben wurden. Daher konnten nicht alle Konzepte systematisch sowohl auf einer objektiven als auch auf einer subjektiven Ebene erfasst werden, z. B. die Betroffenheit von Segregation. Zweitens muss bei der Interpretation der deskriptiven Ergebnisse berücksichtigt werden, dass bestimmte, von Exklusion potenziell betroffene Personengruppen für Umfragen kaum zu erreichen sind. Dazu gehören beispielsweise Strafgefangene, Obdachlose und die Bewohner von Heimen. Darüber hinaus könnte vermutet werden, dass auch in ihren Wohnungen lebende, sozial isolierte Menschen für Befragungen schwerer zu erreichen sind. Könnte man diese Personengruppen befragen, würde möglicherweise ein höherer Anteil von Personen resultieren, die sich als exkludiert wahrnehmen (Schnell 1991). Wir haben uns drittens in unserem Beitrag in der Interpretation unserer Ergebnisse möglichst keiner kausalanalytischen Sprache bedient, sondern überwiegend deskriptive Aussagen über das Vorkommen von Exklusionswahrnehmungen in bestimmten Gruppen und die Kovariation des Exklusionsempfindens mit anderen Einschätzungen der Lebenslage vorgenommen, wobei wir allerdings das Exklusionsempfinden immer als abhängige Variable im Blick hatten. Dies ist auch für einige der untersuchten Variablen durchaus plausibel, so ist schwer vorstellbar, wie das Exklusionsempfinden die Staatsangehörigkeit oder das Alter beeinflussen könnte. Allerdings muss berücksichtigt werden, dass die psychologischen Konsequenzen von sozialer Exklusion zum Teil so gravierend sein können, dass tatsächlich Prozesse denkbar sind, die von sozialer Exklusion zu verstärkter sozialer Isolation, beruflichem Misserfolg und daraus resultierender Armut führen könnten. Insofern wäre das Exklusionsempfinden ein Konzept, das in vorhandene Längsschnittdatensätze aufgenommen werden sollte, um seine langfristigen Einflussgrößen und kausalen Wechselwirkungen zu bestimmen.

## Footnotes

1 Dies basiert auf einer Auswertung des Social Science Citation Index, die für die Kombination der Begriffe *Social* und *Exclusion* eine nahezu lineare Steigerung der Anzahl von Publikationen zwischen 1980 und 2020 anzeigt.

2 Für alternative, informative Analysen der Verwendung des Exklusionsbegriffs siehe Ludwig-Mayerhofer (2009).

3 Die rätoromanische Sprache ist zwar als vierte Landessprache der Schweiz institutionalisiert. Da es sich hier aber um eine sehr kleine Gruppe handelt, ist diese nur begrenzt mithilfe von landesweiten Umfragen zu erforschen, sodass wir diese Gruppe hier nicht berücksichtigen.

4 Insbesondere die angelsächsische Forschung zu diesem Thema knüpft stärker an klassentheoretische Konzepte an, hier insbesondere an den Begriff der Underclass (Welshman 2013).

5 Eigene Berechnungen auf der Basis des Schweizer Haushaltspanels. Das Einsamkeitsgefühl wurde auf einer 11-stufigen Skala von 0–10 abgefragt. Für diese Auswertung wurden die Werte von 6–10 aufaddiert.

6 Die Ausnahme stellt hier der Jurakonflikt dar, der 1979 zur Gründung des Kantons Jura geführt hat.

7 Wir unterscheiden hier zwischen Personen, die mindestens Teilzeit erwerbstätig sind und jenen, die zum Zeitpunkt der Umfrage ohne Arbeit sind, hauptsächlich als Hausfrau/-mann tätig sind, sich in der Schule, Ausbildung oder im Ruhestand befanden.

8 Die Relevanz der Erwerbstätigkeit konnte nicht geprüft werden, da das Prozessmodul von Hayes keine dichotomen Mediationsvariablen zulässt.
